# Morphological Study of the Effect of Aerobic Exercise on Organs and Arteries in Spontaneously Hypertensive Rats

**DOI:** 10.3390/healthcare9081066

**Published:** 2021-08-19

**Authors:** Yingzhe Xiong, Yisheng Luan, Bing Zhang, Shu Zhang, Xiaofei Wang

**Affiliations:** 1 College of Physical Education and Sports, Beijing Normal University, Beijing 100084, China; xyz19@mails.tsinghua.edu.cn; 2 Division of Sports Science and Physical Education, Tsinghua University, Beijing 100084, China; luanys19@mails.tsinghua.edu.cn (Y.L.); bzhang@tsinghua.edu.cn (B.Z.); ychen18@mails.tsinghua.edu.cn (S.Z.)

**Keywords:** aerobic exercise, hypertension, morphology, heart, kidney, arteries

## Abstract

Hypertension is usually accompanied by the impairment of organs and arteries, and seriously threatens human health. Aerobic exercise can effectively prevent and treat hypertension. However, the mechanism of exercise therapy in hypertension is still unclear. In this study, we explored how aerobic exercise effectively reversed the impairment of the heart, kidney, and arteries caused by hypertension through a pathomorphological perspective. Spontaneously hypertensive rats were subjected to fifteen weeks of 45 min and 90 min swimming training without weight, and we then tested the effect of exercise on the morphology and structure of the heart, kidney, iliac artery, and branch of the mesenteric artery. We found that the myocardial fibers became thinner, the cross-sectional area of myocardial cells decreased, and cardiomyocyte edema disappeared after 45 min of aerobic exercise. Additionally, the pathological microstructure of glomeruli and renal tubules were improved. At the same time, aerobic exercise could also reverse the morphology and structure of arteries and mesenteric artery branches in spontaneously hypertensive rats.

## 1. Introduction

Hypertension is one of the most complex diseases in the world, characterized by increased arterial blood pressure with pathologic changes in arterial structure. This can cause atherosclerosis in the arteries and obstruct blood flow by narrowing the lumen of the arteries. In clinical practice, hypertension is divided into two main categories: primary or essential and secondary hypertension. The former is an independent disease in which elevated blood pressure is the main clinical manifestation and the etiology is not yet clear, accounting for more than 90% of all hypertensive patients. In contrast, the latter has a clear etiology, and hypertension is only one of the clinical manifestations of the disorder, such as hypertension of renal parenchyma. The range of blood pressure in hypertensive patients is generally defined as ≥140 mmHg and diastolic blood pressure ≥ 90 mmHg, which is generally consistent with the range of hypertension in rats. Moreover, the heart and kidney are the commonly damaged targets of hypertension. It has been realized that the control of blood pressure alone is not enough for hypertensive patients. Efforts should also be aimed at protecting target organs and arteries to reduce the morbidity and mortality of relative complications [1].

Left ventricular hypertrophy (LVH) is one of the most common target organ damages in hypertension [2]. It is an adaptive change to a prolonged increase in hemodynamic load, manifested by wall thickening, myocardial remodeling, and increased myocardial weight [3]. The remodeling process of LVH involves many aspects, such as neurohumoral factors and cytokines [4]. The kidney, as the main organ regulating the balance of body fluid and electrolyte, is not only an important organ for blood pressure regulation, but also one of the main target organs damaged by hypertension [5,6]. The pathogenesis of kidney damage may be related to oxidative stress and inflammation, hemodynamics, and regulation of the production of vasoactive factors [7]. The arteries are an important place for blood flow, providing nutrients and oxygen to the body’s tissues. In patients with hypertension, the walls of the arteries are under greater pressure, causing damage to the arteries by making them stiffer and less compliant [8,9].

Since the 1980s, exercise has been recommended as a nonpharmacological method to treat hypertension [10]. In 2007, the American College of Sports Medicine proposed that “Exercise is medicine” [11]. Currently, aerobic exercise can help to prevent and treat hypertension and is widely accepted and valued [12]. A recent study showed that aerobic exercise could enhance the vasodilation of the mesenteric artery and reduce blood pressure by inhibiting the PKCα/Ca^2+^ channel pathway in rats [13]. Swimming exercise could alleviate renal dysfunction in rats caused by hypertension by reducing renal interstitial fibrosis [14]. However, for cardiovascular and renal patients, it is unclear whether exercise can reverse the pathological morphological structure and reduce the risk factors for cardiovascular disease.

In this work, we studied the effects of aerobic exercise on the morphological indica-tors of the heart, kidney and arteries of spontaneously hypertensive rats (SHRs) and concluded that aerobic exercise six times a week for 15 weeks can significantly improve some pathological forms of hypertension, which provides a more scientific basis for exercise therapy for hypertension.

## 2. Materials and Methods

### 2.1. Experimental Animals

Male spontaneously hypertensive rats (SHRs) and Wistar rats, aged 12 weeks, were purchased from Charles River (Beijing, China). SHRs are commonly used models of spontaneous hypertension in rats with a systolic blood pressure range of generally 150 mmHg to 200 mmHg, whereas the mean systolic blood pressure in Wistar rats used as controls is generally 126 mmHg. The sample size calculations were based on the method in this literature [15] (α = 0.05; power = 0.9) and combined with the indicator of blood pressure (σ = 15.67; δ = 23.26) in the previous related study [14]. It was estimated that each group needed 8 rats. All rats were housed in ventilated cages at controlled temperature (22 ± 2 °C) and humidity (50 ± 5%) with a 12 h light–dark cycle and free access to water and food. All experiments were performed in compliance with the protocol approved by the Institutional Animal Care and Use Committee (IACUC; Assurance Identification Number: F16-00228; A5061-01) of Tsinghua University. After acclimating to their environment for one week, the rats were randomly divided into four groups (*n* = 8 per group): normal control (Wistar, NC), sedentary (SHR, SC), short training (SHR, ST), and long training (SHR, LT). Swimming training without weights was then performed.

### 2.2. Exercise Protocols

The rats in the ST and LT groups were familiarized with swimming for one week with the swim duration starting with 5 min and increasing by 10 min each day, and then trained for 15 weeks (starting at 9 am, 6 days per week). The ST groups swam for 45 min per day, while the LT groups swam for 90 min per day. The water temperature was 35 ± 2 °C.

### 2.3. Experiment Design and Sample Collection

**Figure 1:**
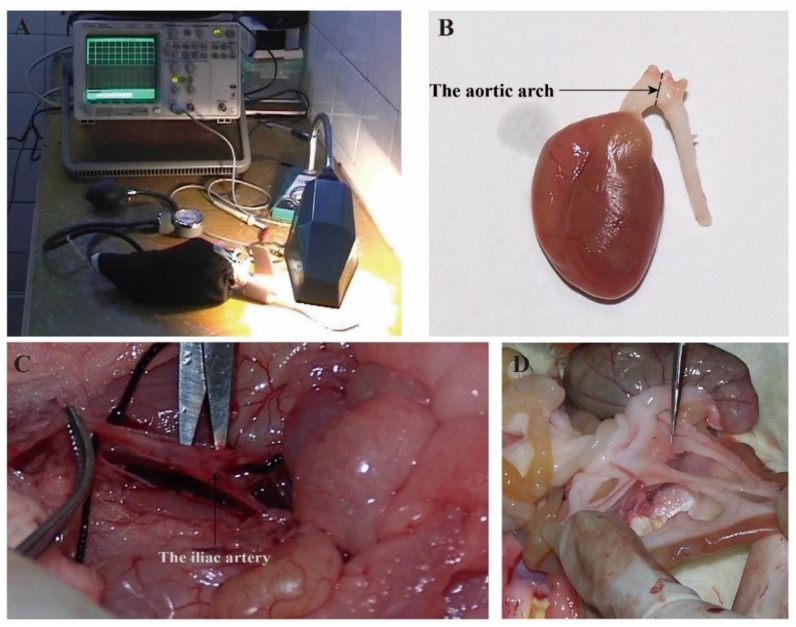
Taking Samples. (**A**) Measuring systolic blood pressure of the caudal artery in rats; (**B**) physical images of the aortic arch; (**C**) physical images of the iliac artery; (**D**) physical images of the branch of mesenteric artery.

Before the 15 week training experiment, the body weight (BW) of the rats was measured using an electronic balance. Additionally, we used an electric light to partially heat the rat tail until the skin turned a reddish color and then tested systolic pressure by a rat blood pressure meter (HX-II, Changsha, China). After the last training experiment, the rats were allowed to rest for 24 h, and then the BW and systolic pressure of the caudal artery in the rest and awake states were measured, as shown in Figure 1A. Next, we anesthetized the rats and cannulated them in the direction of circulation through the proximal end of the renal artery above the branch. Ten percent formalin was perfused and fixed in the body at 90 mmHg pressure for 60 min, and then we removed the heart, kidney, and arcus aortae (the three branches of the innominate artery, left common carotid artery, and left subclavian artery). We took the specimen approximately 2 mm from the proximal end of the innominate artery, vertical to the long axis of the vessel (see Figure 1B), and the iliac artery (the two branches of the right and left iliac arteries). We took the specimen approximately 2 mm from the beginning of the right iliac artery bifurcation of the dorsal aorta, vertical to the long axis of blood vessels (see Figure 1C), and the branch of mesenteric artery (we carefully separated the secondary branch of mesenteric artery approximately 2 mm from the proximal pylorus 8–10 cm through the colon, see Figure 1D).

### 2.4. Hematoxylin and Eosin (H&E) Staining

The heart (coronal view of the middle left ventricle), kidney (coronal view of the middle left kidney), arcus aortae, iliac artery, and branch of the mesenteric artery were excised, washed with ice-cold PBS (Merck, Kenilworth, NJ, USA), and fixed in 10% formalin. After being dehydrated in a graded alcohol series and embedded in paraffin wax, sections of tissue (thickness of 4 μm) were prepared and stained with H&E for histopathology, visualized by a Motic DMBA 450 microscope (200×), imaged by E200 Nikon 950, and then analyzed by Motic Image Advanced 3.1 software (these pictures were analyzed by Yingzhe Xiong and Yisheng Luan). We analyzed the media thickness (MT), the media area (MA), the lumen area (LA), and the lumen diameter (LD). We then calculated the ratio of MT/LD, MA/LD, and MA/LA.

### 2.5. Statistical Analysis

All data are expressed as the mean ± standard deviation (SD) and were analyzed with the Statistical Package for the Social Sciences version 11.0 (SPSS 11.0, IBM, Armonk, NY, USA). In all statistical comparisons, one-way ANOVA was used along with Tukey or Bonferroni post hoc tests to determine statistical differences between experimental group means. *p* values less than 0.05 were considered to indicate statistically significant differences.

## 3. Results

### 3.1. Aerobic Exercise Improved Body Weight and Systolic Blood Pressure

In this study, SHRs were trained to swim without weight. The exercise type (aerobic exercise) and frequency (six times per week) of the two exercise groups was the same, while the exercise duration was different. Our results showed that the body weight (SC vs. ST: *p* < 0.001, Cohen’s d = 3.013; SC vs. LT: *p* < 0.001, Cohen’s d = 3.205) and systolic blood pressure (SC vs. ST: *p* = 0.002, Cohen’s d = 1.687; SC vs. LT: *p* = 0.03, Cohen’s d = 1.020) of the two exercise groups were significantly lower than those of the SC group after 15 weeks of swimming training (Table 1).

### 3.2. Aerobic Exercise Improved Heart Morphology and Structure

**Figure 2:**
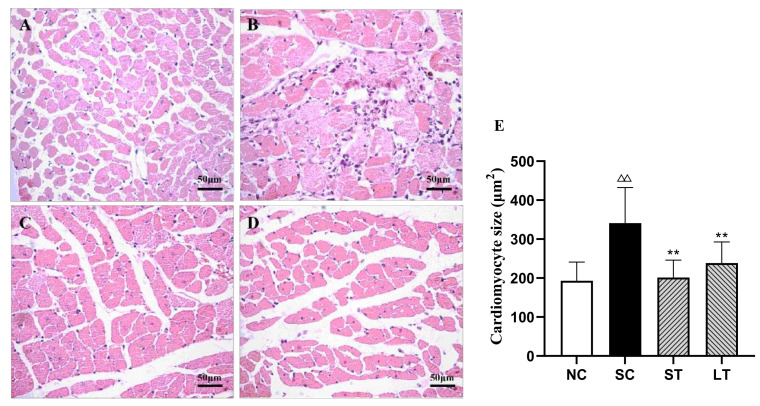
Cardiomyocyte. NC group (**A**), SC group (**B**), ST group (**C**), LT group (**D**), and size of cardiomyocytes (**E**). Data were expressed as the mean ± SD (*n* = 8 rats/group). ^ΔΔ^ *p* < 0.01 compared with NC; ** *p* < 0.01 compared with SC.

**Figure 3:**
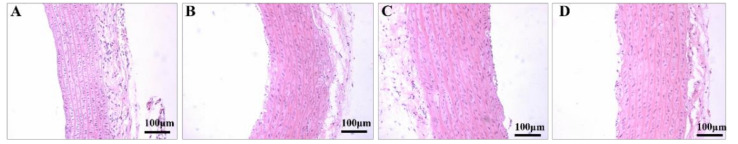
The aortic arch. NC group (**A**), SC group (**B**), ST group (**C**), and LT group (**D**).

Left ventricular hypertrophy (LVH) is a chronic adaptive mechanism of the heart to the long-term persistent load of hypertension [16]. Comparing images of the hearts of the study groups with the NC group, the SC group showed that myocardial fibers thickened, the cross-sectional area of myocardial cells increased, myocardial cells loosened and were stained with edema, and inflammatory cells increased but had no myocardial fibrosis. The size of cardiomyocytes was improved significantly in both exercise groups (SC vs. ST: *p* < 0.001, Cohen’s d = 1.939; SC vs. LT: *p* < 0.001, Cohen’s d = 1.359) (Figure 2). However, 15 weeks of swimming training did not have a positive effect on the structure of the aortic arch (SC vs. ST: *p* = 0.925, Cohen’s d = −0.762; SC vs. LT: *p* = 0.908, Cohen’s d = −0.698) (Figure 3, Table 2).

### 3.3. Aerobic Exercise Improved Kidney Morphology and Structure

**Figure 4:**
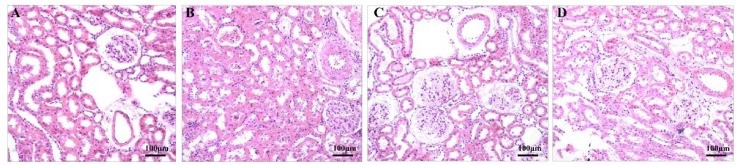
The kidney. NC group (**A**), SC group (**B**), ST group (**C**), and LT group (**D**).

Hypertension damages the renal vasculature and impairs the function of the kidney [17]. Compared with the NC group, the SC group showed that the walls of small arteries in the kidney were thickened, the lumens of the small arteries and proximal convoluted tubules were narrowed, the endothelial cells of the proximal convoluted tubules were turbid and swollen, and the number of VSMCs (vascular smooth muscle cells) and cells in the glomeruli were increased (Figure 4). The abovementioned structures improved in both training groups (MT/LD SC vs. ST: *p* < 0.001, Cohen’s d = 3.288; SC vs. LT: *p* < 0.001, Cohen’s d = 2.608. MA/LD SC vs. ST: *p* < 0.001, Cohen’s d = 3.004; SC vs. LT: *p* < 0.001, Cohen’s d = 2.935. MA/LA SC vs. ST: *p* < 0.001, Cohen’s d = 2.920; SC vs. LT: *p* < 0.001, Cohen’s d = 2.408.), with the ST group better improved than the LT group (MT/LD LT vs. ST: *p* = 0.029, Cohen’s d = 1.028; MA/LA LT vs. ST: *p* = 0.043, Cohen’s d = 0.924.) (Table 3).

### 3.4. Aerobic Exercise Improved Artery Morphology and Structure

**Figure 5:**
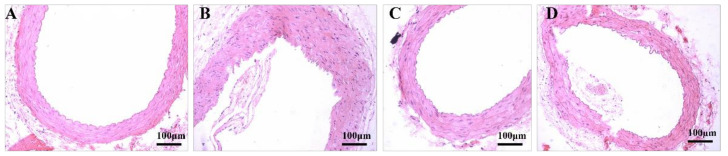
The iliac artery. NC group (**A**), SC group (**B**), ST group (**C**), and LT group (**D**).

**Figure 6:**
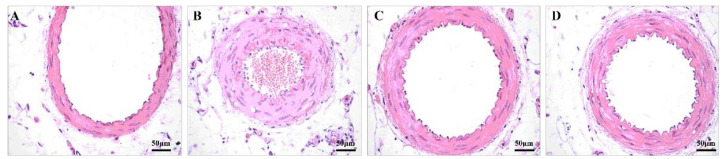
The branch of mesenteric artery. NC group (**A**), SC group (**B**), ST group (**C**), and LT group (**D**).

Pathological changes in arterial morphology are not only the basic pathological changes of hypertension, but also the morphological basis for maintaining high vascular resistance and further development and deterioration of hypertension [18]. For the iliac artery, the microscopy images showed that, in the SC group, the iliac artery wall was thickened and the lumen was narrowed, the structure of intermuscular elastic fibers was blurred, the arrangement of media VSMCs was disordered, and the number of VSMCs was increased when compared with the NC group (Figure 5). After exercise, the iliac artery of the ST group improved significantly (MT/LD SC vs. ST: *p* =0.018, Cohen’s d = 1.167. MA/LD SC vs. ST: *p* = 0.020, Cohen’s d = 1.131. MA/LA SC vs. ST: *p* = 0.019, Cohen’s d = 1.146.). Meanwhile, the LT group did not show the similar therapeutic effect (MT/LD SC vs. LT: *p* =0.9875, Cohen’s d = −1.256. MA/LD SC vs. LT: *p* = 0.1584, Cohen’s d = 0.519. MA/LA SC vs. LT: *p* = 0.9858, Cohen’s d = −1.222.) (Table 4). For the branch of the mesenteric artery, images showed that, compared to the NC group, the mesenteric artery branch wall was thickened, the lumen was narrowed, the number of medial VSMCs was increased, and the density of endothelial cells was increased and was protruding into the lumen in the SC group (Figure 6). The abovementioned structures improved in the ST group and the LT group (MT/LD SC vs. ST: *p* = 0.014, Cohen’s d = 1.226; SC vs. LT: *p* = 0.026, Cohen’s d = 1.066. MA/LD SC vs. ST: *p* =0.096, Cohen’s d = 0.685; SC vs. LT: *p* = 0.198, Cohen’s d = 0.439. MA/LA SC vs. ST: *p* =0.018, Cohen’s d = 1.154; SC vs. LT: *p* = 0.032, Cohen’s d = 1.006.) (Table 5).

## 4. Discussion

Hypertension is a major risk factor for heart failure, angina pectoris, myocardial infarction, renal failure, and other cardiovascular diseases. Its high incidence and mortality have seriously threatened human health and aroused extensive attention in the medical field [19]. Exercise therapy has been widely confirmed to treat hypertension, and its mechanisms include neurohormonal regulation, such as lowering serum catecholamine levels associated with lower total peripheral resistance [20]. Aerobic exercise also lowers blood pressure through its effect on blood vessel function [21]. Our study demonstrated that aerobic exercise for 15 weeks, 45 min a day, could effectively reverse pathological morphological and structural changes in the heart, kidney, and arteries. This provides direct histomorphological evidence for aerobic exercise in the treatment of hypertension.

Left ventricular hypertrophy (LVH) is one of the most characteristic changes among the pathological changes of hypertensive ventricular morphology and structure. Studies have shown that the reversal of LVH is closely related to the reduction in the risk of cardiovascular diseases in the course of hypertension [22,23,24]. According to a recent clinical meta-analysis, the effective reversal of LVH can reduce the risk of cardiovascular diseases in subsequent hypertension patients [25]. Previous studies have shown that exercise can moderately upregulate protein kinase B, improve myocardial metabolism through downstream mTOR (mammalian target of rapamycin, mTOR), protect cardiac functions, and delay cardiac hypertrophy [26,27]. Our results show that the myocardial fibers became thinner, the cross-sectional area of myocardial cells decreased, and cardiomyocytes edema disappeared after 45 min of aerobic exercise, effectively improving LVH. This mechanism may be that exercise promotes the secretion of interleukin-6 (IL-6) and inhibits tumor necrosis factor α (tumor necrosis factor-α, TNF-α), IL-1 β, and other proinflammatory factors, so as to play an anti-inflammatory role. A clear conclusion requires further research [28].

The kidney is one of the most important target organs impaired by hypertension, and kidney injury is manifested as renal arterioles damage, such as renal hemodynamics and morphological changes, which cause an increase in resistance vessels [29,30]. Many studies have found that aerobic exercise can improve renal hemodynamics and is beneficial to the treatment of hypertension [31,32]. Exercise has been reported to significantly inhibit glomerular degeneration in renal cortical tissue and hypertension-induced tubular degeneration, cell clustering, and tubular cell swelling as well as to attenuate interstitial fibrosis and renal cell apoptosis [33]. The results of this study showed that 45 min of aerobic exercise significantly improved the pathological microstructure of glomeruli and renal tubules, as well as renal arterioles with a lumen diameter of 50~100 μm, in which the MT/LD, MA/LD, and MA/LA in the exercise group were significantly lower than those in the sedentary group. This may be attributed to the fact that exercise affects vascular remodeling by modulating the renin-angiotensin system [34].

The pathological change in arterial structure in the early stage of hypertension is an adaptive response to maintain the tension of the vascular wall. However, during the development and maintenance of hypertension, pathological changes in vascular structure cause pathological changes in the heart, brain, kidney, and other target organs [35,36]. Hypertension is also an important factor promoting the occurrence of cardiovascular complications with high mortality. The improvement of pathological changes in arterial structure in hypertension has become a new direction for antihypertensive therapy. The most typical pathological changes in arterial structure during hypertension include thickening of the middle layer, decreasing the inner diameter, and increasing the stroma thickness [37]. Our study found that 45 min aerobic exercise could significantly improve the morphology and structure of the iliac artery and branches of the mesenteric artery, which indicated that aerobic exercise improved hemodynamics by changing the morphology and structure of the renal vasculature. These results are consistent with earlier reports. The remodeling of the vasculature by exercise includes the enlargement of artery diameter and the thinning of the tube wall. Exercise also improves endothelial dysfunction and increases elastin content, thereby restoring normal arterial function [38,39]. Additionally, in this study, we found that an exercise duration of 45 min was more effective in improving physiological morphological indices of hypertension than of 90 min. This may be caused by some exercise damage to the blood vessels due to prolonged repetitive exercise, and in-depth studies are still needed to obtain definite conclusions. Therefore, exercise duration is a factor that must be taken into account when performing exercise interventions in hypertensive patients.

## 5. Limitations and Future Directions

### 5.1. Possible Mechanism of Aerobic Exercise to Improve Hypertension

The present study provides an experimental basis for the histomorphology of exercise in the treatment of hypertension, while little has been said about its specific mechanism. In our study, we found that exercise reduces inflammatory infiltration due to hypertension, and whether this is related to morphological changes in target organs. In-depth studies can be conducted to address these questions in terms of inflammatory factors and related pathways.

### 5.2. Exercise Prescription for Hypertension

In this study, we examined the effect of two exercise durations, 45 and 90 min, on the improvement of histomorphology of the main targets of hypertension. Whereas this is not sufficient to establish an exercise prescription for hypertension, the effects of factors such as exercise form, exercise intensity, exercise frequency, and exercise duration need to be further investigated in order to better guide clinical practice.

## 6. Conclusions

In summary, our study demonstrated that aerobic exercise could not only effectively reduce blood pressure, but also effectively reverse cellular damage to target organs and arteries by hypertension from the perspective of morphology, which indicates that the influence of exercise on morphological and structural changes in organs and arteries should also be regarded as an important aspect in exercise therapy for hypertension. In addition, aerobic exercise of 45 min duration was significantly better than 90 min in the treatment of hypertension, which provides a scientific basis for establishing an exercise prescription for hypertension.

## Figures and Tables

**Table 1:** Body weight and systolic blood pressure.

Group	Body Weight (g)		Systolic Blood Pressure (mmHg)	
	Pre-Training	Post-Training	Pre-Training	Post-Training
NC	429.60 ± 26.93	542.60 ± 43.85	126.17 ± 12.51	107.40 ± 5.37
SC	258.50 ± 10.52 ^Δ^	353.00 ± 17.09 ^ΔΔ^	181.73 ± 16.26 ^ΔΔ^	187.50 ± 17.62 ^ΔΔ^
ST	257.63 ± 10.14	301.63 ± 17.01 **	176.17 ± 16.19	159.88 ± 15.02 **
LT	252.38 ± 14.69	288.00 ± 23.03 **	181.29 ± 16.59	168.94 ± 18.77 *

Data were expressed as the mean ± SD (*n* = 8 rats/group). ^Δ^ *p* < 0.05 and ^ΔΔ^ *p* < 0.01 compared with NC; * *p* < 0.05 and ** *p* < 0.01 compared with SC.

**Table 2:** The media thickness of the aortic arch after training.

Group	MT (μm)
NC	101.241 ± 22.493
SC	142.312 ± 18.581 ^ΔΔ^
ST	166.147 ± 40.119
LT	161.022 ± 33.056

Data are expressed as the mean ± SD (*n* = 8 rats/group). ^ΔΔ^ *p* < 0.01 when compared with NC.

**Table 3:** Morphological parameters of intrarenal arterioles after training.

Group	MT/LD	MA/LD	MA/LA
NC	0.211 ± 0.033	48.970 ± 10.316	1.027 ± 0.186
SC	0.391 ± 0.092 ^ΔΔ^	105.399 ± 27.825 ^ΔΔ^	2.207 ± 0.673 ^ΔΔ^
ST	0.143 ± 0.054 **	36.915 ± 16.286 **	0.665 ± 0.324 **
LT	0.197 ± 0.051 **^#^	44.046 ± 9.988 **	0.953 ± 0.299 **^#^

Data were expressed as the mean ± SD (*n* = 8 rats/group). ^ΔΔ^ *p* < 0.01 compared with NC; ** *p* < 0.01 compared with SC; ^#^ *p* < 0.05 compared with ST.

**Table 4:** Morphological parameters of the iliac artery after training.

Group	MT/LD	MA/LD	MA/LA
NC	0.217 ± 0.056	368.075 ± 64.693	1.069 ± 0.317
SC	0.227 ± 0.026	411.249 ± 72.320 ^Δ^	1.119 ± 0.154
ST	0.189 ± 0.038 *	342.216 ± 47.191 *	0.904 ± 0.216 *
LT	0.265 ± 0.034 ^##^	382.047 ± 33.168 ^#^	1.342 ± 0.207 ^##^

Data are expressed as the mean ± SD (*n* = 8 rats/group). ^Δ^ *p* < 0.05 compared with NC; * *p* < 0.05 compared with SC; ^#^ *p* < 0.05 and ^##^ *p* < 0.01 compared with ST.

**Table 5:** Morphological parameters of the branch of mesenteric artery after training.

Group	MT/LD	MA/LD	MA/LA
NC	0.193 ± 0.120	149.974 ± 108.107	1.046 ± 0.731
SC	0.383 ± 0.117 ^ΔΔ^	161.956 ± 49.207	2.170 ± 0.863 ^ΔΔ^
ST	0.242 ± 0.113 *	130.511 ± 42.309	1.251 ± 0.723 *
LT	0.267 ± 0.100 *	141.565 ± 43.618	1.386 ± 0.686 *

Data are expressed as the mean ± SD (*n* = 8 rats/group). ^ΔΔ^ *p* < 0.01 compared with NC; * *p* < 0.05 compared with SC.

## Data Availability

The data presented in this study are available on request from the corresponding author. The data are not publicly available due to privacy reasons.
